# Small fire refugia in the grassy matrix and the persistence of Afrotemperate forest in the Drakensberg mountains

**DOI:** 10.1038/s41598-017-06747-2

**Published:** 2017-07-26

**Authors:** Hylton Adie, D. Johan Kotze, Michael J. Lawes

**Affiliations:** 1 School of Life Sciences, University of KwaZulu-Natal, P/Bag X01, Scottsville, 3209 South Africa; 2 Department of Environmental Sciences, University of Helsinki, Niemenkatu 73, FIN-15140 Lahti, Finland

## Abstract

Afrotemperate forests situated in the Drakensberg mountains of South Africa are characteristically small (1–10 s ha) and widely dispersed in a vast fire-prone grassland. Compared with lowland forests, they are typically species poor with low levels of endemism and species turnover, patterns that are to date unexplained. Here we show that the richness, composition and functional traits of tree species distributed on extremely small (10–100 s m^2^) rocky fire-refugia situated in grassland are indistinguishable from that in forest. Afrotemperate forest tree species in the Drakensberg are widely dispersed and conform to the habitat generalist strategy. Most forest trees are bird dispersed; wind dispersal is rare and is associated only with species that resprout in response to fire. We present the ‘matrix refuge hypothesis’, which proposes that fire and extreme conditions associated with exposed rocky outcrops have filtered the Afrotemperate forest tree composition resulting in convergence in functional traits essential for trees to arrive, establish and persist on fire refugia in the grassland matrix. Most Afrotemperate forest tree diversity in the Drakensberg thus resides in the matrix where it may function as a recolonisation reservoir during climatic bottlenecks.

## Introduction

High elevation (>1400 m a.s.l.) Afrotemperate^1^ forests distributed in the Drakensberg mountains of South Africa are generally small and discrete, located on south-facing slopes and in steep-sided valleys, and embedded within a vast grassy matrix^2^. In this grassland matrix, woody plant establishment is suppressed by grass competition and fire^3, 4^. In addition, ‘edge’ habitat is a dominant component of these forests due to their small size^5^. The tree flora of Afrotemperate forests is greatly impoverished compared with lowland forests, has low levels of endemism and displays surprising uniformity and homogeneity^6, 7^. In contrast, the surrounding grassland matrix is floristically rich with high levels of endemism^3, 7^. Their scattered distribution, isolation and small size beg the question how and why these montane forests persist in this grass-dominated and fire-prone landscape. Here we examine the proposition that the long and intimate association with the surrounding fire-prone grassland matrix^3, 7^ has shaped the composition, diversity and functional traits of trees occurring in montane forests of eastern South Africa.

Forest tree species of the Afrotemperate landscape also persist on extremely small (10–100 s m^2^) fire-protected topographic refugia situated in the grassy matrix. The refuge sites are located up-slope of large rocks, on rocky outcrops and above cliff-lines on isolated grassy spurs. Although these sites are protected from fire, they are exposed to extreme conditions that characterise the grassland matrix. The proliferation of pioneer tree species in the matrix is a common trend in fragmented forest landscapes^8, 9^. In addition, the non-random mortality of mature trees exposed in fragments and the differential survival among regenerating trees causes functional convergence on the ruderal life-history among species in edge and matrix habitats^10, 11^. Because a pool of colonising ruderal species persists in the matrix, refuge sites may have a profound influence on the structure and composition of habitat fragments^9, 12^. Understanding why these ruderal species persist in refuge sites – essentially what plant functional traits confer this persistence and what environmental filters select for these traits – may provide novel insights to understanding the composition, diversity and history of establishment of forest in this mountainous region.

Forest tree establishment and survival in the matrix are strongly filtered by physical conditions. Temperature, soil moisture and light change substantially across the forest-matrix boundary^13^ and typically causes a decline in shade-tolerant tree species through regeneration failure^12, 14, 15^. Grass competition and fire are key constraints to tree regeneration in the grassland matrix^16^. In general, tree species that establish and persist in a grassland matrix share the characteristics of good colonisers with a ruderal life-history; they are light-tolerant, relatively fast growing and tolerate exposure to regular disturbance^17^. In addition, seed rain in the matrix is strongly skewed towards small-seeded pioneer tree species^18^. Large-seeded tree species, while often capable of establishing in successional environments^16^, are usually absent due to dispersal limitation^19^. In contrast, small-seeded trees often have multiple dispersal vectors, many of which are not affected by fragmentation or do not avoid disturbed sites^17, 20^. Thus, we predict that in the fire-prone Afrotemperate landscape, conditions imposed by the grassland matrix on the establishment of woody plants have a pervasive effect on the composition of forests that establish there.

We examine the role of the grassy matrix in shaping forest tree composition, diversity and functional traits in a montane landscape where forests are naturally small and have a patchy distribution. We evaluate the proposition that the forest tree assemblage associated with refuge sites in the matrix is a non-random subset of the assemblage found in forest^14, 21^. Because of the dual filtering effects of fire and the severe conditions associated with exposed rocky sites, we predict that the forest tree assemblage on extremely small topographic refugia should comprise fire-adapted generalist and/or ruderal species^8, 9^ - the ‘matrix-refuge hypothesis’. We address the following questions:

1. Does the cumulative species richness, diversity and composition of forest trees distributed among extremely small matrix refugia (10–100 s m^2^) differ from forest (1–10 s ha)?
2. Are species-poor refuge sites subsets of species-rich sites - representing a deterministic assemblage composition of tree species in fire-refugia in the matrix? Because fire has a greater effect on smaller than larger refugia, forest tree species may only occupy larger, less exposed refuge sites. In contrast, if fire-adapted ruderal species dominate the tree assemblage then they will occupy all refuge sizes, and tree species occupancy of fire refugia in the matrix will not be nested.
3. Do functional traits of the tree assemblage persisting in extremely small refugia differ from forest? We predict convergence of functional traits among tree species on traits representative of a ruderal life strategy due to the filtering effects of fire and exposure in the matrix^8, 10^. In addition, diaspores of species in refugia should be bird dispersed because this is the most reliable and targeted mode of seed dispersal to sites within a generally inhospitable matrix^22^.

## Results

**Figure 1:**
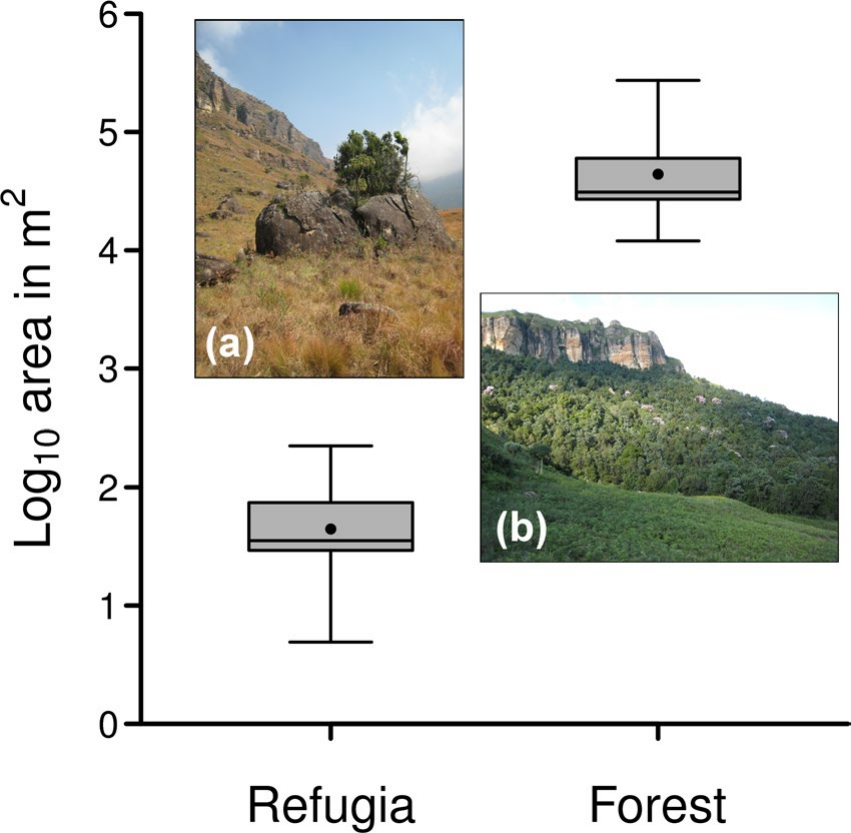
A box plot illustrating the size (log_10_ of area in m^2^) distribution of topographic refugia (n = 39) and forest (n = 5) at Royal Natal. Inset photographs show (**a**) forest trees assembled within a rocky fire-refuge site surrounded by grassland and (**b**) Afrotemperate forest on a south-facing slope situated beneath a sandstone ridge at Royal Natal, the principal study site. Error bars represent extreme measurements. Images by Hylton Adie.

Topographic refugia varied considerably in size (mean: 62 m^2^; median: 35 m^2^; range: 5–224 m^2^, n = 39) and were at least three orders of magnitude smaller than forest (Fig. 1, Supplementary Figs S1–S3). Refugia were associated with rocky sites, large boulders or stream banks and were surrounded entirely by grassland. The mean distance from refugia to the nearest forest was 320 m (range: 30–730 m, n = 39).

### Diversity indices

**Figure 2:**
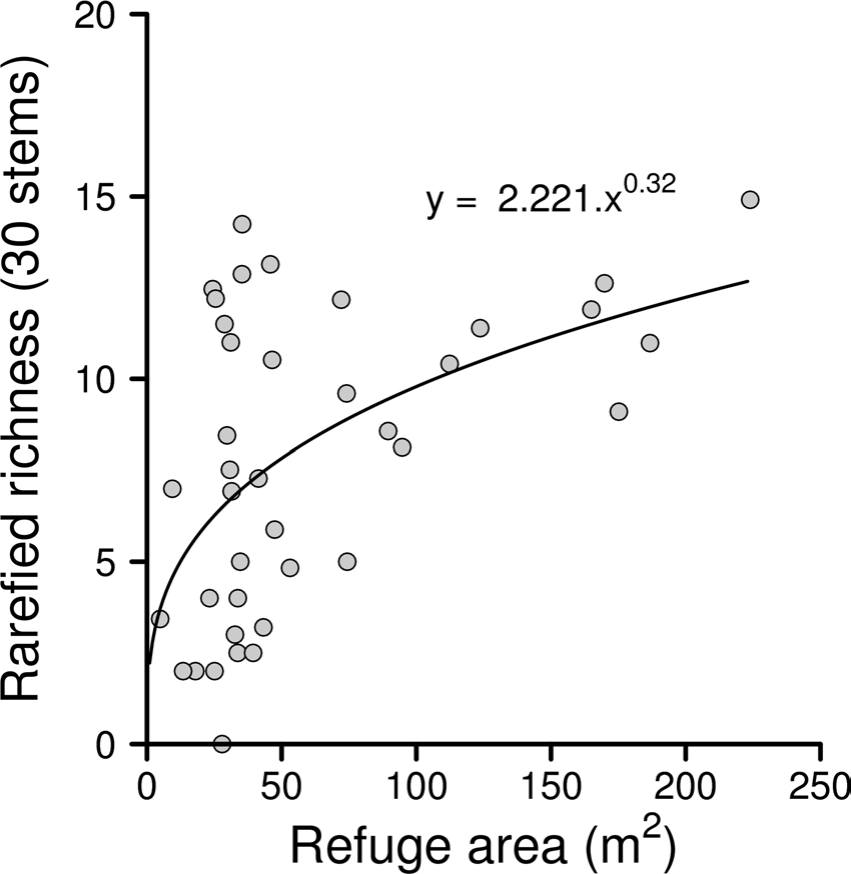
Relationship between species richness of forest trees recorded in topographic refugia and size of refugium. Richness estimates were extrapolated or rarefied to 30 stems.

We recorded 37 woody species on topographic refugia (Table 1, Supplementary Table S4). Twelve of these tree species, hereafter referred to as refuge-only species, are generally not associated with mature forest but are common components of the forest ecotone and incipient forest. Correcting for stem density, the number of forest tree species recorded from refugia increased with refugium size (Fig. 2). However, the relationship was relatively weak because several refugia in the 30–50 m^2^ range were richer in species than sites several-fold their size. The ten most abundant species recorded from refugia accounted for 68% of individuals (1064) and nine of these were common to forest. Notably, *Podocarpus latifolius*, a shade-tolerant forest tree, was the third most abundant species recorded from refugia. *Searsia dentata*, the second most abundant tree on refugia, is a resprouting tree absent from mature forest but common to the forest ecotone.

**Table 1:** The number of observed species (S_obs_), rarefied species richness (95% CI) and species diversity measured by the Shannon exponential (95% CI) for 39 refuge sites and five forests at Royal Natal.

Site	S_obs_	S_ref_	Species richness	Species diversity
Refugia (all species)	37		31 (27–34)	24 (23–26)
Refugia (forest species only)	25		23 (20–26)	15 (14–16)
Gudu forest (27.3 ha)	23	18	23 (22–25)	10.6 (9.0–12.2)
Devil’s Hoek forest (6.0 ha)	24	19	—	9.0 (7.7–10.1)
Tendele forest (3.1 ha)	15	11	—	7.9 (6.4–9.5)
Thugela Gorge forest (2.7 ha)	12	11	—	4.2 (3.0–5.3)
Thugela river forest (1.2 ha)	10	10	—	5.1 (4.1–6.1)
Combined forests	29	21	25 (22–28)	11.2 (10.3–12.2)

S_ref_ refers to the number of tree species recorded from forest that were also present on refugia. The number of species was rarefied to 376 individuals, the number of individuals recorded from Gudu forest. Shannon exponential is measured in units of equally abundant species and is thus directly comparable between groups. The 95% confidence interval was calculated using a bootstrap method (200 iterations). Combined forests represents the combined samples (n = 35) for the five forests. Species richness estimates by rarefaction were not calculated for the smaller forests because of the low numbers of trees sampled.

**Figure 3:**
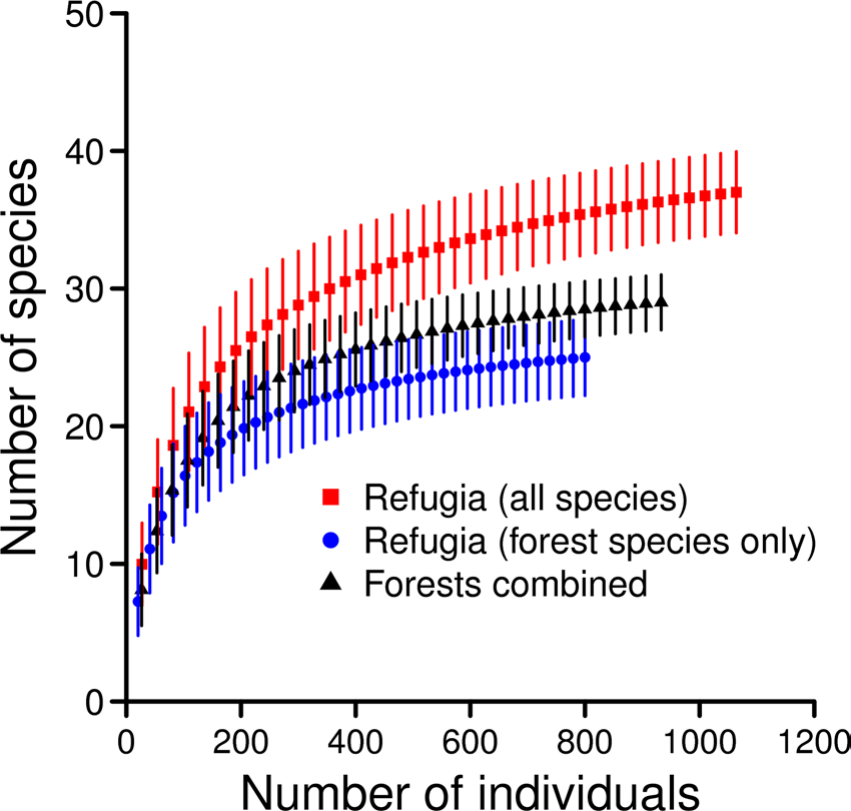
Sample-based rarefaction curves, adjusted for different numbers of individuals, for woody tree species recorded from grassland topographic refugia and forest (five forests combined). Tree species not recorded in forest were removed (i.e. forest species only) from the second refuge curve. Each point represents the mean of 100 randomisations of sample pooling and error bars are 95% confidence intervals. The area sampled in forest was ~3.6-fold greater than the combined sampled area of refuge sites.

The area sampled in forest was ~3.6-fold greater than the combined sampled area of refuge sites. We recorded 29 tree species from the five forests combined, and 23 tree species from the largest forest (Table 1). In contrast, 25 forest tree species were recorded from refuge sites and 76% of all forest tree species reported were recorded from refugia. Tree species richness and diversity on refuge sites were significantly higher than in forest (Table 1, Fig. 3). However, when controlling for stem density, forest tree richness on refugia was indistinguishable from that in Gudu forest (largest forest, 27.3 ha) and all forests combined. The rarefaction curve for tree species in topographic refugia did not asymptote at 39 sites (Fig. 3) indicating that more tree species are likely in the grassland matrix. The diversity of forest tree species in refugia was noticeably higher compared with the forest habitats (Table 1).

The proportion of forest species in forest that was also recorded from refugia varied from 72% (combined forest assemblage) to 100% (Tugela river forest, Table 1). Although eight forest species were not recorded from refugia, four forest tree species that were absent from our combined forest sample, were recorded on refuge sites. Both canopy (75% of all species) and mid-canopy (77% of all species) trees were recorded from refuge sites (Supplementary Table S4).

### Nestedness

Tree species composition on refugia was not nested either for all tree species combined or when refuge-only species were removed (Supplementary Table S5). The observed NODF values for these tree assemblages did not deviate from random communities generated by either the PP or FF null models.

### Tree functional traits and species distribution

**Figure 4:**
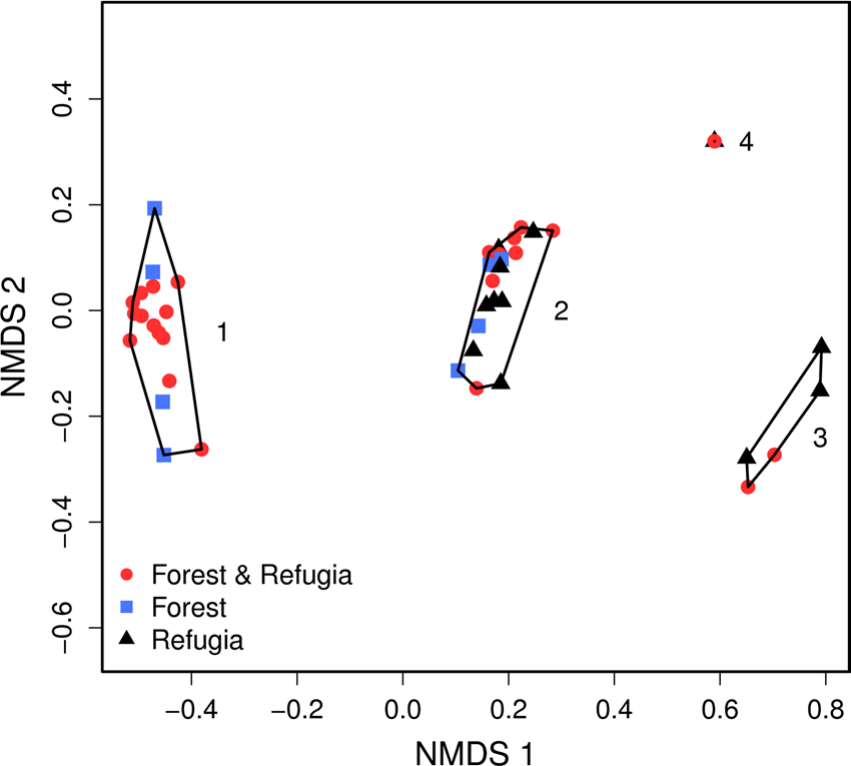
NMDS ordination of 37 tree species based on Bray–Curtis species similarity coefficients for six functional traits. Axis 1 is a combination of dispersal mode and the capacity to resprout. Three clear groups were identified: Group 1 (n = 18): bird dispersed non-sprouters; Group 2 (n = 20): bird-dispersed resprouters; Group 3 (n = 5) wind-dispersed resprouters. *Cussonia* spp. (4) are bird dispersed resprouters characterised by low WD, low SLA and thick bark compared with species assigned to group 2. Forest & Refugia: species recorded from forest and refuge sites; Forest: species recorded from forest only; Refugia: species recorded from refuge sites only. Stress = 0.065.

The NMDS identified three clear functional groups on axis 1 defined by dispersal mode and the capacity to resprout after fire (Fig. 4): (1) bird dispersed non-sprouters; (2) bird dispersed resprouters; and (3) wind-dispersed resprouters. *Cussonia spicata*, a bird dispersed resprouter, was separated from group 2 because this species is characterised by low WD, low SLA and thick bark when compared with the mean trait values of group 2. Forest tree species were recorded from all three groups. In contrast, tree species confined to refuge sites resprouted in response to fire.

The REML model demonstrates a significant difference in functional traits among habitats, dispersal mode and between resprouting responses to fire (Table 2). Bark thickness differed significantly among habitats (forest-only, forest and refuge, refuge-only) and between fire responses (resprouter, non-sprouter) but not among dispersal modes (bird vs. wind dispersal) (Tables 3 and 4). Bark thickness measured at 6 cm bole diameter, was thinnest in forest and thickest on refugia. Bark was almost twice as thick in resprouters compared with non-sprouters. Although bark was thicker on average in wind dispersed species than in bird dispersed species, this difference was not significant. Even though non-sprouting forest species (group 1) typically had thin bark, they nevertheless were distributed on refuge sites. Seven of the eight most fire-sensitive species, as measured by bark thickness, were recorded on refugia. Tree species confined to refuge sites were wind or bird dispersed and all resprouted in response to fire disturbance. In contrast, 56% (n = 25) of forest tree species recorded from topographic refugia lacked a clear fire-tolerance mechanism.

**Table 2:** Multivariate restricted maximum likelihood (REML) linear mixed model results for bark thickness, diaspore size, specific leaf area and wood density with dispersal mode, resprouting response to fire and habitat as fixed effects.

Fixed term	*df*	*F*	*P*
Dispersal mode	4, 77.7	11.97	<0.001
Resprouting response	4, 77.7	4.41	<0.01
Habitat	8, 95.1	3.63	<0.01

All of the trait response variables were ln-transformed to improve homoscedasticity.

**Table 3:** Restricted maximum likelihood linear (REML) mixed model analysis results for bark thickness (BT_6_), diaspore size (D_size_), specific leaf area (SLA) and wood density (WD) with dispersal mode, resprouting response to fire and the habitat in which species were recorded as fixed effects.

Source of variation	*df*	BT_6_		D_size_		SLA		WD	
		*F*	*P*	*F*	*P*	*F*	*P*	*F*	*P*
Dispersal mode	1,40	2.49	0.122	43.13	**<0.001**	3.37	0.074	0.12	0.727
Resprouting response	1,40	15.46	**<0.001**	0.55	0.462	0.11	0.739	1.96	0.169
Habitat	2,40	9.13	**<0.001**	1.90	0.163	2.65	0.064	1.0	0.377

Bold type indicates significant effect at P < 0.05.

**Table 4:** Mean (95% CI) bark thickness (BT_6_), diaspore size (D_size_), specific leaf area (SLA) and wood density (WD) and results of the univariate F-tests summarised for the fixed effects of the REML model (habitat, dispersal mode, resprouting response to fire).

	Habitat			
	Forest & Refuge (25)	Forest (8)	Refuge (12)	F-Test
BT_6_ (mm)	2.9 (2.1–4.0)	2.0 (1.5–2.4)	5.6 (3.6–7.6)	*F*_2,40_ = 9.13, ***P*** < **0.001**
D_size_ (mm)	7.6 (6.2–9.6)	10.1 (6.9–14.3)	6.8 (4.5–8.8)	*F*_2,40_ = 1.93, *P* = 0.163
SLA (m^2^ kg^−1^)	9.6 (8.1–11.4)	12.6 (9.9–14.7)	12.3 (9.2–16.1)	*F*_2,40_ = 2.94, *P* = 0.064
WD (g cm^−3^)	0.60 (0.57–0.64)	0.62 (0.57–0.66)	0.56 (0.48–0.63)	*F*_2,40_ = 1.00, *P* = 0.377
	Dispersal mode			
	Bird (40)	Abiotic (5)		
BT_6_ (mm)	3.1 (2.4–3.9)	6.2 (2.9–9.5)		*F*_1,40_ = 2.49, *P* = 0.12
D_size_ (mm)	8.6 (7.5–10.1)	2.0 (1.0–3.4)		*F*_1,40_ = 43.12, ***P*** < **0.001**
SLA (m^2^ kg^−1^)	11.2 (9.9–12.7)	7.9 (7.3–8.4)		*F*_1,40_ = 3.37, *P* = 0.074
WD (g cm^−3^)	0.60 (0.57–0.63)	0.56 (0.47–0.64)		*F*_1,40_ = 0.12, *P* = 0.727
	Fire Response			
	Non-resprouter (18)	Resprouter (27)		
BT_6_ (mm)	1.8 (1.4–2.2)	4.6 (3.5–6.0)		*F*_1,40_ = 15.46, ***P*** < **0.001**
D_size_ (mm)	8.8 (7.1–10.7)	7.2 (5.6–8.9)		*F*_1,40_ = 0.55, *P* = 0.462
SLA (m^2^ kg^−1^)	10.2 (8.7–11.6)	11.3 (9.5–13.3)		*F*_1,40_ = 0.11, *P* = 0.739
WD (g cm^−3^)	0.63 (0.59–0.66)	0.57 (0.52–0.61)		*F*_1,40_ = 1.96, *P* = 0.169

Forest & Refuge: species recorded from forest and refuge sites; Forest: species recorded from forest only; Refuge: species recorded from refuge sites only. The 95% confidence interval was calculated using a bootstrap method (200 iterations). Sample size is in brackets after fixed effect treatment. Bold type indicates significant effect at P < 0.05.

More than 95% of forest tree species recorded from topographic refugia and forest were vertebrate-dispersed and of these, all were bird dispersed (Supplementary Fig. S6). We recorded two wind-dispersed forest species and both resprout in response to fire. Diaspore size did not differ significantly among habitats or between fire response types, but was significantly smaller in wind dispersed species than bird dispersed species (Tables 3 and 4). Forest species tended to have larger diaspores on average. However, of the vertebrate-dispersed forest trees, >90% of species had diaspores of intermediate size (Supplementary Fig. S6).

SLA did not differ significantly in any of the fixed terms. There was a marginal tendency towards significant variation among habitats and dispersal modes. WD did not differ significantly in any of the fixed terms (Table 3).

The analyses of plant functional traits revealed that: (1) there are clearly defined functional groups among tree assemblages at our study site; (2) most forest trees, including the most fire-sensitive species, occur on refuge sites; 3) tree species that are confined to forest do not appear to be functionally different from forest species that occur on refuge sites; but 4) trees confined to the grassland matrix (i.e. refuge-only species) require the capacity to resprout and thick bark to persist in this fire-prone environment.

The distribution of most forest tree species recorded in this study extends well beyond South Africa with 67% occurring in equatorial Africa (Supplementary Fig. S6). Approximately 80% of forest tree species were distributed over the entire elevation gradient from the inland mountains to the coast, a differential of ~1950 m. Eighty-six percent of forest tree species have an equatorial distribution or are recorded from coastal KwaZulu-Natal and were thus considered habitat generalists.

There was strong phenotypic clustering among species in topographic refugia as demonstrated by significant correlations between species co-occurrence and phenotypic distance matrices. Species that were good post-fire resprouters tended to co-occur in the fire-affected refugia (*r* = 0.86, *P* < 0.001, Mantel test). These results confirm the importance of fire as an ecological filter of tree species found in these montane forests.

## Discussion

In spite of a 1000-fold size differential, we found no difference in the accumulated forest tree richness of refugia situated in the grassland matrix compared with that in forest. The composition and functional traits of forest trees on refuge sites were indistinguishable from those in forest, and most trees were widely distributed in Africa or over an elevation gradient of almost 2000 m in South Africa. Non-forest tree species that were confined to refugia were all resprouters and possessed thick bark, functional traits that confer persistence in fire-prone ecosystems. However, fire-sensitive forest trees were also common on refuge sites underlining the critical role of refugia to their persistence in the matrix. Tree species confined to forest were not functionally different from those forest species recorded from refuge sites. Apart from the conifer *Afrocarpus falcatus*, which is marginally shade-tolerant, these species are light demanding and regarded as pioneer or forest edge species indicating that their absence from refugia is likely to be a sampling effect. There was no evidence of nestedness of tree species composition among refugia indicating random establishment by forest tree species, and implying that forest tree species in this system are functionally similar. As predicted, there was a preponderance of bird-dispersed species associated with refuge sites. In contrast, wind-dispersed forest tree species, which survive fire events by basal resprouting, were uncommon at our Drakensberg study site. Our results indicate functional convergence (biotic homogenization^23^) or the phenotypic clustering of traits^24^ essential for trees to establish and persist on small fire refugia in the matrix. This proliferation of generalist tree species in the matrix under environmental filtering is consistent with results from fragmentation studies in tropical forest ecosystems^9, 14, 15^.

Species converge on similar trait syndromes that permit persistence under the most limiting environmental constraint^25, 26^. Conditions characteristic of the matrix exert an overwhelming filtering^27^ effect on the composition of fragmented biotas^21^. For example, fire and extreme drying events combine in fragmented forest environments to cause widespread tree mortality^28, 29^. In southern Africa, the antiquity of floristically rich grasslands points to a long association between forest and grassland, and one where fire has had an influential role in shaping the composition of woody trees^3, 7, 30^. The spread of C_4_ dominated grasslands in Africa, dated to the Late Miocene/Pliocene, was associated with increasing aridity and a rise in fire activity^31^. The subsequent fire-driven ‘orgy of extinction’^1^ of montane tree floras is likely to have been further reinforced by the repeated climatic oscillations of the Pleistocene^32–34^. We propose that these filtering events would have favoured: (1) directional selection for bird dispersal to topographic refugia; and (2) trait convergence among tree species for tolerance to fire disturbance and conditions typical of exposed sites. We argue that forest tree species of the Afrotemperate region have thus, paradoxically, converged on a suite of life history traits that favour persistence in the matrix. This has resulted in a forest flora dominated by a robust suite of generalist species, with broad climatic envelopes^35^, that are conveniently resilient to contemporary anthropogenic disturbance. It is thus not surprising that Afrotemperate tree floras show remarkable uniformity and homogeneity^6, 7^.

The critical importance of birds in a grass-dominated landscape is clearly demonstrated by the asymmetric arrival of bird-dispersed tree species to nuclei situated in the matrix^22^. Thus, tree species that are killed by fire nevertheless persist in the fire-prone matrix through directed dispersal by birds to fire-safe refugia. The prevalence of medium-sized dispersal vectors in fragmented environments selects preferentially for tree species with small to medium-sized diaspores^20^, a finding consistent with ours at Royal Natal, where more than 40% of bird species recorded from forest are omnivorous (M.J. Lawes, unpublished data). In contrast, the unpredictable outcome of wind-dispersal is unlikely to be retained in fire-prone environments. Indeed, wind-dispersed seeds rarely find their way to wooded nuclei situated in the matrix^22^. Wind-dispersed forest tree species are conspicuous by their absence at Royal Natal but, as expected, those present survive regular burning episodes by basal sprouting.

While fire is a strong species filter, the most fire-sensitive tree species nevertheless persist in fire-safe topographic refugia suggesting that environmental variables other than fire are important in determining the forest species pool on refuge sites. Drought-induced tree mortality is accelerated on exposed sites and in shallow, rocky soils^36^. Forest trees growing on refuge sites, where high temperatures, wind and shallow soils combine to increase moisture stress, are thus especially vulnerable to drought^37^. However, most forest tree species in our study appear to tolerate these conditions. Apart from deciduousness^38^ (27% of forest species) we find little direct evidence of functional traits (e.g. high wood density^36, 39^) that enhance survival under drought conditions. In fact, the absence of species with high wood density (>0.8 g cm^−3^) suggests that the benefits conferred by mechanical strength or cavitation resistance at the expense of growth rate^39, 40^ places trees at a selective disadvantage in the Afrotemperate environment. Indeed, growth rate negatively correlates with wood density and drought mortality; and light-demanding species typically have low wood density^36, 39^. Competition for light favours rapid growth among light-demanding trees^41^ and rapid growth confers fire resistance as bark thickness increases with diameter growth in fire-prone environments^42^. Thus, there is immediate selective advantage for life-history traits that promote rapid juvenile growth among the light-demanding trees that dominate Drakensberg forests^2^. We propose that the stressful conditions that characterise refuge sites, filter out specialists (e.g. slow growing shade-tolerant species) leaving a suite of generalists that grow fast and tolerate drought.

*Podocarpus latifolius* (Podocarpaceae), which is widespread and common in Drakensberg forests, is an enigma in this environment because it is shade-tolerant and slow growing. It is surprising that the conifer persists in this mountainous landscape because shade-tolerant species are susceptible to fragmentation^14, 15^. However, *P. latifolius* is unusual among shade-tolerant species in that it regenerates successfully in open environments. Indeed, this species is frequently the most conspicuous component of topographic refugia in the Drakensberg (Supplementary Fig. S3). Slow growth is thus offset by shade tolerance when competing with fast growing light-demanding forest flora.

The persistence of forest tree species on extremely small refugia confers resilience to altered fire regimes that may arise through climate change. The matrix may therefore function as a reservoir for forest tree species during climatic bottlenecks such as those that punctuated the Pleistocene^32–34^. A forest species pool that resides in the matrix eliminates the need for long-distance dispersal across extensive grasslands to re-colonise these mountainous regions following climate-induced contraction or fire-driven loss of forest. Indeed, only those tree species that tolerate the high exposure conditions characteristic of rocky refugia persist in this mountainous landscape, resulting in a generally impoverished forest tree flora dominated by ruderal species.

Historical environmental filtering has had a pervasive influence on the contemporary ecology of forests in this mountainous region. For example, in the absence of stand-levelling disturbance, Afrotemperate forest develops inexorably towards a low-diversity state dominated by shade-tolerant conifers^2, 43^, a situation that arises from conifer longevity coupled with the failure of angiosperm canopy species to regenerate beneath an intact canopy^44^. Afrotemperate forest diversity is thus maintained at the landscape scale by catastrophic disturbance in the form of crown fire in primary forest^45^. A subtle outcome of the matrix-refuge hypothesis presented here is that forest habitat *per se* is not necessary for the persistence of forest tree species in this mountainous landscape. In fact, most forest trees need a fire-free environment beyond the confines of forest to establish because they fail to recruit beneath a closed canopy. The grassy matrix, dominated by shade-intolerant C_4_ grasses, is thus especially vulnerable to invasion by these broadly-adapted tree species when fire is withheld^2, 3, 46^.

Laurance *et al*.^21^ predicted that fragmented ecosystems would become increasingly impoverished and dominated by matrix-tolerant generalists and disturbance-adapted opportunists. This is precisely the pattern that characterises Afrotemperate forest in eastern South Africa^6, 7^. Africa is a continent of grass, fire and drought^30, 33^, a combination of factors deadly to tree species distributed in naturally small forests persisting in grass-dominated environments. We propose that repeated filtering events, which started with the proliferation of C_4_ grasslands in the Late Miocene, and exacerbated by recurring climatic bottlenecks, have shaped the tree composition of forested mountainous environments in southern Africa.

## Methods

### Study region

We compared tree species richness and diversity in five montane forests (1.2–27 ha) with that in 39 small grassland topographic refugia at Royal Natal, a conservation area within the uKhahlamba Drakensberg Park World Heritage Site, KwaZulu-Natal province, South Africa (Supplementary Figs S1–S3). Forests in the northern Drakensberg are small (mean = 2.2 ha, median = 0.8 ha, n = 1490, Adie & Lawes unpublished data) and are typically tucked beneath rocky sandstone belts of south-facing slopes. Royal Natal was selected as a study site because it provides the widest range of forest sizes in the Drakensberg, enabling us to compare tree composition in the largest forests with that in refuge sites. The extent of the largest forest (Gudu; 27.3 ha) is exceptional for the Drakensberg (within the top 0.5 percentile of all forests, n = 2339). The size of the remaining four forests (range: 1.2–6.0 ha, Table 1) is typical for the region. Human-altered burning regimes are known to favour the encroachment of woody plants into grassland^47^. We avoided this potential confounding effect by selecting forest and topographic refuge sites (also referred to as refugia) that were surrounded entirely by primary grassland. The grasslands at Royal Natal have not been grazed by domestic stock since the reserve was proclaimed in 1916. Forests in the Heritage Site are not actively managed and no form of harvesting is permitted. Topographic refuge sites were sampled on a south-east facing grassland slope (~9°), which is burned on a 3-year cycle.

### Diversity indices

Forest trees were sampled in five forests at Royal Natal using a modified Whittaker plot method^2^. Plots of 25 m × 10 m were randomly positioned in forests, and trees >10 cm diameter at breast height (d.b.h.) were recorded. The number of plots sampled varied from 3–14 according to forest size. We sampled the forest edge and interior (>30 m from the edge) to capture the full range of forest stand structure and tree species composition. We recorded all established woody plants in topographic refugia. Trees growing in refugia are smaller than those in forest with few exceeding 10 cm d.b.h. We therefore defined established plants as taller than 50 cm or, failing that, sexually mature (fruit/flower present). Fire-safe refugia are generally positioned alongside, and between, large rocks and on rocky outcrops. The area of refuge sites was calculated as πLW/4, where L is the longest axis and W the longest orthogonal width. We placed an upper size limit of 225 m^2^ (i.e. ~ 15 × 15 m) on topographic refugia.

Tree species richness in refuge sites was compared with forest using Mao-Tau sample-based rarefaction^48^, adjusted for unequal numbers of individuals. We compared tree species diversity between forests and refugia using a non-parametric estimator of Shannon entropy that adjusts for unseen species^49^. Diversity is presented as units of equally abundant species^50^ calculated from the exponential of Shannon entropy^49^.

### Nestedness

Nestedness occurs when the species composition of depauperate assemblages is a true subset of the composition of species-rich assemblages^51^. Only those tree species capable of tolerating fire in the matrix are expected to occur on small refuge sites. In contrast, fire sensitive forest canopy trees should only be present in larger refuge sites. We examined nestedness of tree species assemblages among topographic refugia using the NODF metric (nestedness based on overlap and decreasing fill), an incidence-based measure that is independent of the nestedness matrix properties (e.g. size, fill, shape)^51^. Nestedness was analysed with the NODF program^52^. Two null model algorithms were used to evaluate the departure of the observed NODF metric from random: (1) The fixed-fixed (FF) null model, which preserves column and row totals of the observed matrix but randomises the pattern of species co-occurrence, has traditionally been recommended to detect nestedness because its conservative algorithm reduces Type I errors^51^. (2) More recently, the proportional–proportional (PP) algorithm, where rows and columns vary randomly but the average row and column totals of the random set of matrices match those of the original matrix, has been shown to have greater power than the FF null model in identifying nested assemblages but the algorithm is vulnerable to Type I errors^53^. The use of both algorithms is thus recommended for robust analyses. Significance was evaluated by comparing the observed NODF metric with the expected value generated by the null model (1000 simulations).

### Tree functional traits

Functional traits provide insights into how organisms respond to environmental change^26^. We measured six traits representing functional adaptations to key processes in the Drakensberg montane landscape, four of which were included as response variables (bark thickness, diaspore size, specific leaf area, wood density) in a multivariate REML, while two (dispersal mode, resprouting response to fire) as well as habitat were predictor variables. First, bark thickness and the capacity to resprout determine tree persistence in fire-prone ecosystems^42, 54^. Bark thickness (BT_6_) was measured according to Lawes, *et al*.^42^ and measurements were standardised to a bole diameter of 6 cm for interspecific comparison^55^. Second, we recorded resprouting capacity as a binary trait based on our knowledge of tree species in these montane forests. Tree species were defined as resprouters if they produced basal sprouts in response to fire damage. Third, wood density (WD; ratio of oven-dry mass to wet volume) has been shown to be a surrogate for drought tolerance and growth rate^39, 56^. Wood density was collected following the method in Cornelissen *et al*.^57^. Next, we recorded specific leaf area (SLA; ratio of leaf area to oven-dry mass), a measure of relative growth rate^58^, according to Cornelissen *et al*.^57^. Finally, we selected two regenerative traits, dispersal mode (D_mode_) and diaspore size (D_size_), which reflect dispersal distance and the establishment and survival of seedlings under hostile conditions^59^. We first classified all woody plant species as dispersed by vertebrates or abiotic mechanisms^17^. Vertebrate-dispersed diaspores typically have brightly coloured fleshy pulp or an aril. In contrast, propagules dispersed by abiotic means are usually dull in colour and have appendages that facilitate wind, gravity or ballistic dispersal^59^. We further categorised all vertebrate-dispersed woody species according to the following diaspore sizes^17^: (1) <6 mm in diameter; (2) 6–15 mm; (3) 16–30 mm; and (4) diaspores >30 mm in diameter (i.e. the limiting size for birds that swallow fruit).

Non-metric multidimensional scaling (NMDS) was used to visualize tree species assemblage similarity according to plant functional traits. The Bray-Curtis index was used to calculate the initial pairwise dissimilarities between species. Data were square-root transformed prior to running the NMDS (1000 iterations). The plant functional trait data were further analysed using multivariate restricted maximum likelihood (REML) linear model analysis^60^. Multivariate REML models provide efficient estimates of treatment effects in unbalanced designs with more than one source of error and can be used in situations where one would normally use MANOVA but have unbalanced or correlated data. The usefulness of multivariate REML in our case is that all the response variables (bark thickness, diaspore size, specific leaf area and wood density) can be simultaneously included in the model, which avoids incurring Type I errors. If significant differences are detected in the multivariate model then one proceeds with the univariate tests of the fixed effects. Accordingly, all the available response variables were simultaneously examined with dispersal mode, resprouting response to fire and habitat in which species were recorded as fixed effects. All of the trait response variables were ln-transformed to improve homoscedasticity. All analyses were conducted using GenStat version 18^61^.

We evaluated whether co-occurring species in refugia had similar fire resilience phenotypes (phenotypic clustering) by correlating co-occurrence and phenotypic distance matrices using a Mantel test (999 iterations^24^). Our resilience trait was post-fire resprouting ability^54^. Pairwise values of species co-occurrence in 39 refugia were calculated using the Jaccard binary distance index. The phenotypic distance matrices were computed by calculating the pairwise binary distances between phenotypic states (resprouter or not) of the species. These analyses were conducted using the ade4 package (http://pbil.univ-lyon1.fr/ADE-4/) implemented in R^62^.

### Generalist/specialist classification

To complement the above life history and functional trait data, we classified tree species as habitat specialists or generalists according to their geographic distribution in Africa and their elevational distribution in KwaZulu-Natal province (sea level to ~1950 m). Generalist species tend to occupy large geographical ranges due to their broad climatic tolerance but there are performance trade-offs in any given environment^63, 64^. Thus, we defined habitat generalist tree species as those with ranges that extend to equatorial Africa or have a local distribution that extends to the KwaZulu-Natal coast. In contrast, habitat specialists were defined as species endemic to the montane zone in South Africa. We defined three elevation categories: mountain ~1500–1950 m a.s.l; midlands 900–1500 m a.s.l; coast <100 m a.s.l. Tree species with a distribution restricted to South Africa (including Lesotho or Swaziland) were defined as endemic to South Africa. Species whose distribution was south of the Zambezi River (Namibia, Botswana, Zimbabwe, southern Mozambique) were categorised as ‘southern African’. All remaining species that extend into equatorial Africa were defined as ‘widespread’. Coates Palgrave^65^ was consulted for southern African distributions and we supplemented these data with an online database for the rest of Africa^66^.

### Data Availability

The datasets generated during and/or analysed during the current study are available from the corresponding author on reasonable request.

## Electronic supplementary material

Supplementary Information
