# Supplementary material for: Small fire refugia in the grassy matrix and the persistence of Afrotemperate forest in the Drakensberg mountains
Source: Sci Rep. 2017 Jul 26;7:6549. doi: 10.1038/s41598-017-06747-2 (PMC5529369; doi:10.1038/s41598-017-06747-2)
Supplement: Supplementary file 1 — Supplementary Information [file 41598_2017_6747_MOESM1_ESM.pdf]

Supplementary Information

Small fire refugia in the grassy matrix and the persistence of Afrotemperate forest in the Drakensberg mountains

Hylton Adie[1]*, D. Johan Kotze[2] and Michael J. Lawes[1]

[1] School of Life Sciences, University of KwaZulu-Natal, P/Bag X01, Scottsville 3209, South Africa.

[2] Department of Environmental Sciences, Niemenkatu 73, FIN-15140, University of Helsinki, Lahti, Finland.

*Correspondence should be addressed to H.A. (hylton.adie@gmail.com)

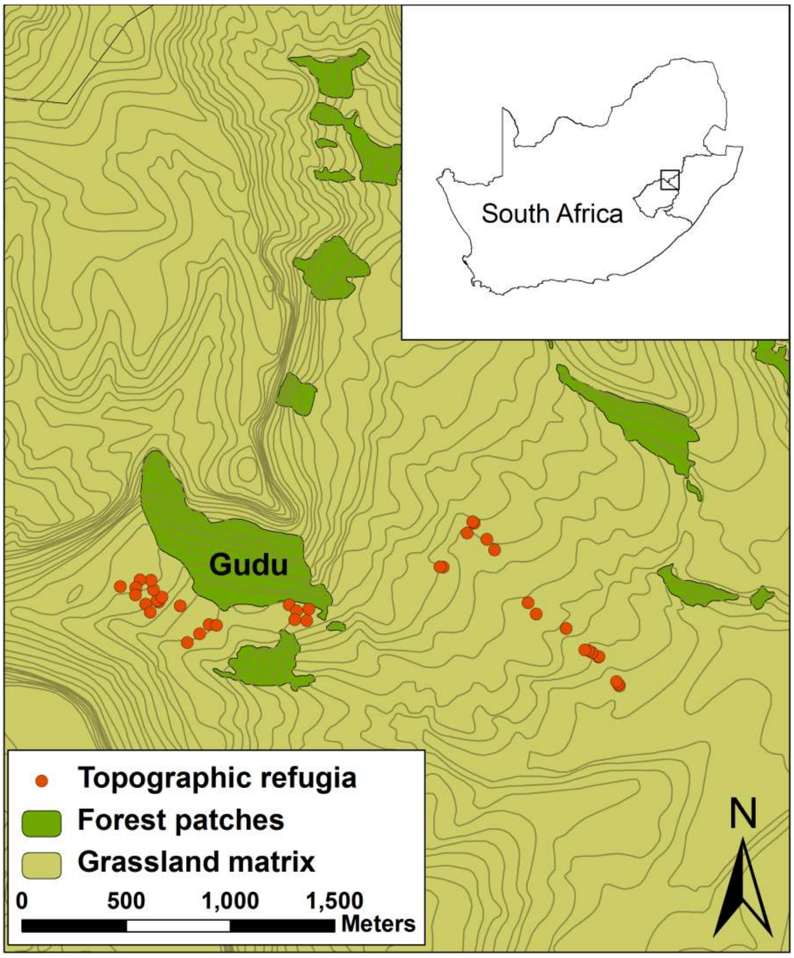

Figure S1. Principal study area showing the distribution of forest and topographic refuge sites sampled at Royal Natal. Gudu forest is 27.3 ha in extent. The contour interval is 20 m. The map was generated with ArcGIS 9.3 (http://www.esri.com).

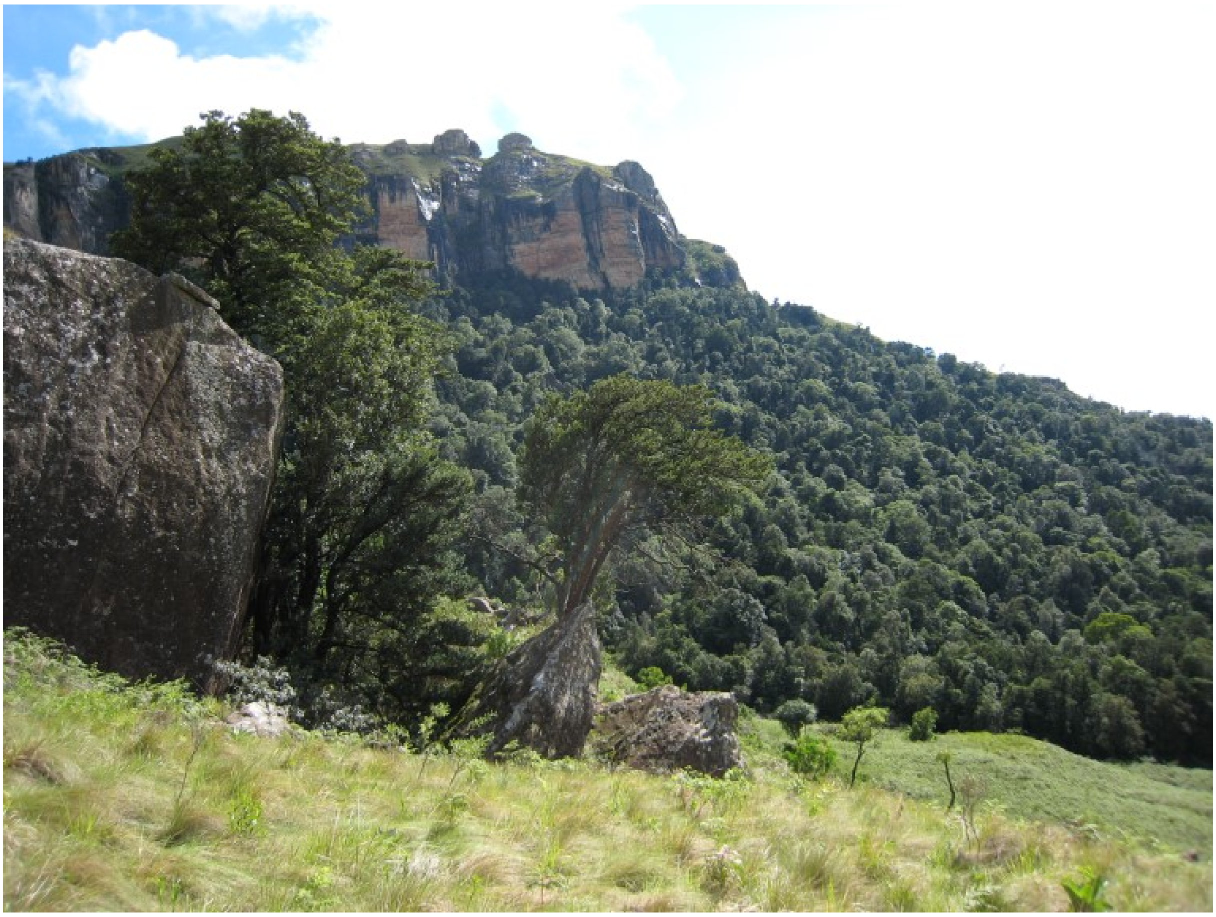

Figure S2. Forest trees in a fire-safe topographic refuge at Royal Natal, the principal study site. Gudu forest is in the background. Image by Hylton Adie.

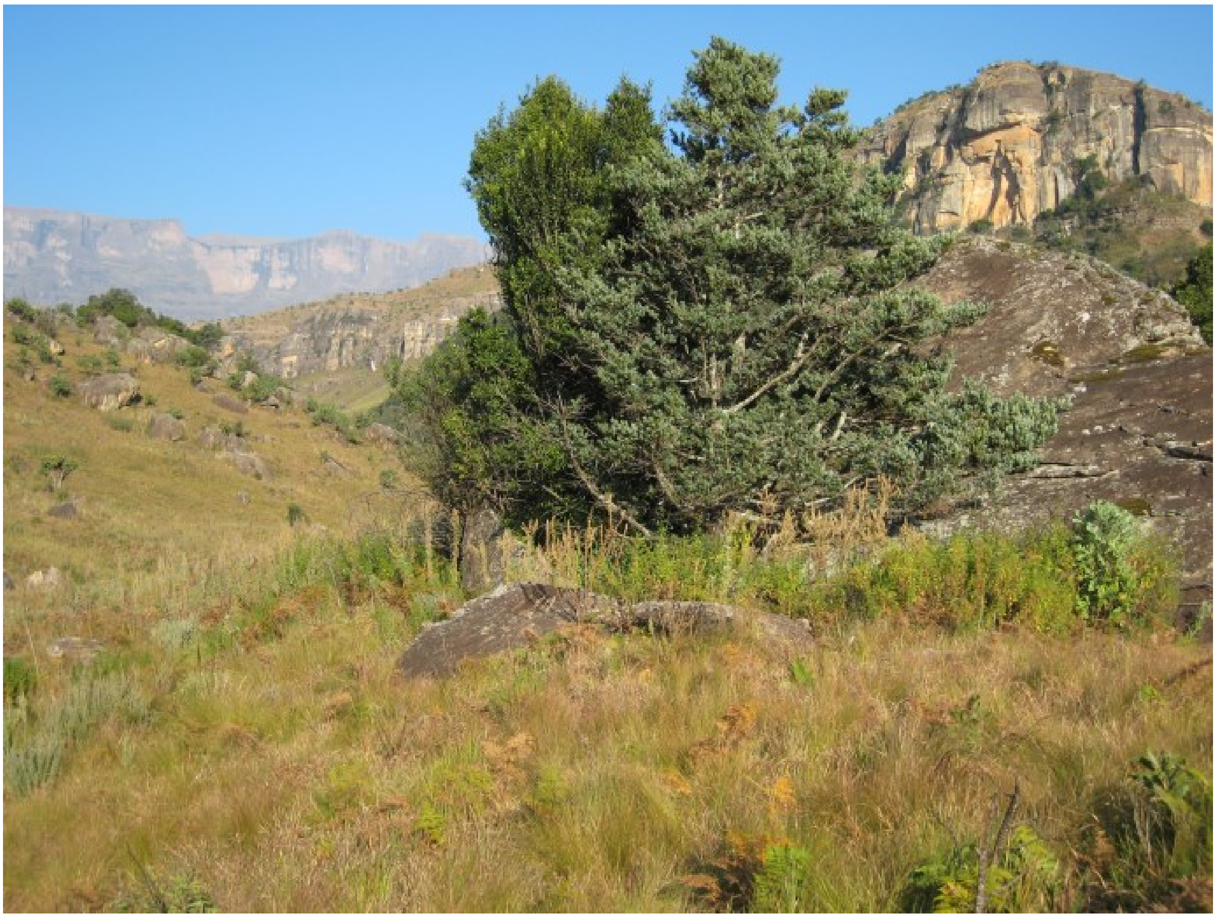

Figure S3. Mature *Podocarpus latifolius* (right) and *Scolopia mundii* (left) persisting on a fire-safe topographic refuge situated in regularly burned grassland at Injasuthi, a conservation area south of Royal Natal, in the uKhahlamba Drakensberg Park World Heritage Site. Image by Hylton Adie.

Table S4. List of tree species recorded from forest and refuge sites at Royal Natal. Species type refers to whether the species occurs in mature forest (F) according to our forest data for the entire Drakensberg region[1]. Species that had not previously been recorded from forest were assigned as refuge (R) species. Distribution refers to whether species were recorded from forest (F), refuge sites (R) or both (F & R) in the present study. Stratum refers to vertical distribution of tree crown: (C – canopy, M – midcanopy, U – understorey). Fire response refers to whether the species resprouts in response to fire disturbance. D_{mode} refers to dispersal mode. Succession refers to the sequence of establishment adapted from a conceptual model of forest successional patterns in the Drakensberg[2]: grassland colonist (fire tolerant); early/edge (fire (in)tolerant); long-lived pioneer (fire intolerant); late succession (shade tolerant conifer). Species names were verified using the iPlant Collaborative Taxonomic Name Resolution Service v4.0[3].

Species	Species type	Distribution	Stratum	Fire response	D_{mode}	Succession
Afrocarpus falcatus	F	F	C	None	Bird	Late succession
Bowkeria verticillata	R	R		Resprouter	Wind	
Buddleja salviifolia	F	F & R	M	Resprouter	Wind	Grassland colonist
Burchellia bubalina	R	R	M	Resprouter	Bird	Early/edge
Calodendrum capense	F	F	C	None	Bird	Long-lived pioneer
Canthium ciliatum	F	F & R	U	Resprouter	Bird	Early/edge
Carissa bispinosa	F	F & R	U	Resprouter	Bird	Early/edge
Cassinopsis ilicifolia	F	F	U	Resprouter	Bird	Early/edge
Celtis africana	F	F & R	C	None	Bird	Long-lived pioneer
Clausena anisata	F	F & R	M	None	Bird	Long-lived pioneer
Cryptocarya woodii	F	F	M	Resprouter	Bird	Long-lived pioneer
Cussonia paniculata	R	R		Resprouter	Bird	
Cussonia spicata	F	F & R	C	Resprouter	Bird	Long-lived pioneer
Diospyros lycioides	R	R		Resprouter	Bird	
Diospyros pubescens	R	R		Resprouter	Bird	
Diospyros whyteana	F	F & R	M	None	Bird	Long-lived pioneer
Ekebergia capensis	F	F & R	C	None	Bird	Long-lived pioneer
Euclea crispa	F	F & R	M	Resprouter	Bird	Early/edge
Grewia occidentalis	R	R	M	Resprouter	Bird	Early/edge
Greyia sutherlandii	R	R		Resprouter	Ballistic	
Gymnosporia buxifolia	F	F & R	U	Resprouter	Bird	Grassland colonist
Halleria lucida	F	F & R	M	Resprouter	Bird	Grassland colonist

Heteromorpha arborescens	F	F & R	M	Resprouter	Bird	Grassland colonist
Ilex mitis	F	F & R	C	None	Bird	Long-lived pioneer
Kiggelaria africana	F	F	C	Resprouter	Bird	Early/edge
Leucosidea sericea	F	R	M	Resprouter	Wind	Grassland colonist
Maytenus acuminata	F	R	M	None	Bird	Early/edge
Maytenus peduncularis	F	F & R	C	None	Bird	Long-lived pioneer
Maytenus undata	F	R	M	None	Bird	Long-lived pioneer
Morella pilulifera	R	R		Resprouter	Bird	
Olinia emarginata	F	F & R	C	None	Bird	Long-lived pioneer
Pittosporum viridiflorum	F	R	C	None	Bird	Early/edge
Podocarpus latifolius	F	F & R	C	None	Bird	Late succession
Protea caffra	R	R		Resprouter	Wind	
Pterocelastrus rostratus	F	F & R	C	None	Bird	Long-lived pioneer
Rapanea melanophloeos	F	F & R	C	Resprouter	Bird	Early/edge
Rhamnus prinoides	F	F	M	Resprouter	Bird	Early/edge
Scolopia mundii	F	F & R	C	None	Bird	Long-lived pioneer
Searsia dentata	R	R		Resprouter	Bird	
Searsia pyroides	R	R		Resprouter	Bird	
Searsia tomentosa	F	F & R	M	Resprouter	Bird	Early/edge
Solanum giganteum	R	R		Resprouter	Bird	
Trimeria grandifolia	F	F & R	M	None	Bird	Long-lived pioneer
Trimeria trinervis	F	F	M	None	Bird	Early/edge
Zanthoxylum davyi	F	F	C	None	Bird	Long-lived pioneer

Table S5. Nestedness based on overlap and decreasing fill (NODF) values of maximally packed matrices for refugia (n = 39) at Royal Natal including (1) all tree species and (2) forest tree species only (i.e. refuge-only species removed). Significance was evaluated by comparing the observed NODF metric with the expected value (simNODF) generated by the null model (1000 simulations) using the one-sided p-value ($P < 0.05$). Observed NODF values were evaluated using the proportional-proportional (PP) and fixed-fixed (FF) null models. The Z-transformed scores and 95% confidence intervals for each simulated distribution are presented.

Site	NODF	simNODF	*Z*	*P*	95% CI
PP null model					
All species	55.04	55.13	-0.04	0.48	50.44–60.29
Forest species	69.05	67.13	0.68	0.25	61.50–72.38
FF null model					
All species	55.04	54.27	0.84	0.20	52.37–55.92
Forest species	69.05	67.85	1.11	0.13	65.51–69.64

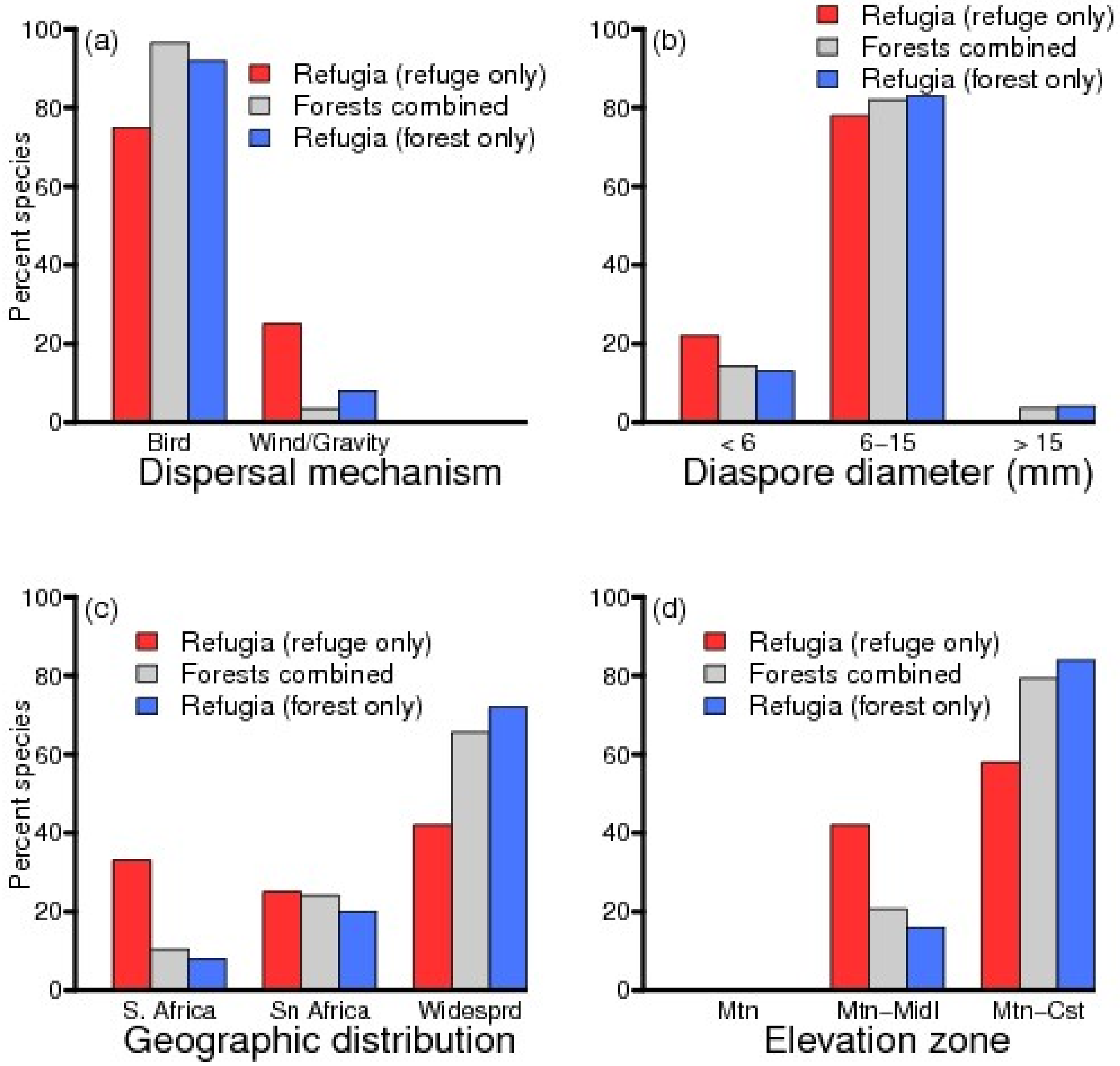

Figure S6. Summary of functional traits and the distribution of tree species recorded from intact forest and refugia at Royal Natal. (a) Dispersal mechanism (bird vs. abiotic). (b) The size distribution of vertebrate-dispersed diaspores. (c) Geographic distribution of forest tree species in Africa. Widespread refers to tree species distributed in East, central or West Africa. (d) Elevation distribution of species in KwaZulu-Natal. Mtn (mountain): ~1500–1950 m a.s.l; Midl (midlands): 900–1500 m a.s.l; Cst (coast): < 100 m a.s.l. Refugia (forest only): forest tree species recorded from refugia; Refugia (refuge only): tree species confined to refugia (i.e. species that do not occur in mature forest); Forests combined: tree species recorded from the five forests at Royal Natal.

References

1 Lawes, M. J., Adie, H., Eeley, H. A. C., Kotze, D. J. & Wethered, R. An assessment of the forests of the Maloti-Drakensberg Transfrontier Bioregion, with reference to important ecosystem processes. (Forest Biodiversity Research Unit, School of Biological and Conservation Sciences, University of KwaZulu-Natal, Pietermaritzburg, 2007).

2 Adie, H. & Lawes, M. J. Role reversal in the stand dynamics of an angiosperm–conifer forest: colonising angiosperms precede a shade-tolerant conifer in Afrotemperate forest. *For. Ecol. Manage.* **258**, 159-168, doi:http://dx.doi.org/10.1016/j.foreco.2009.03.055 (2009).

3 Boyle, B. *et al.* The taxonomic name resolution service: an online tool for automated standardization of plant names. *BMC Bioinformatics* **14**, 1-15, doi:10.1186/1471-2105-14-16 (2013).
